# Paternal—but Not Maternal—Autistic Traits Predict Frontal EEG Alpha Asymmetry in Infants with Later Symptoms of Autism

**DOI:** 10.3390/brainsci9120342

**Published:** 2019-11-26

**Authors:** Valentina Riva, Cecilia Marino, Caterina Piazza, Elena M Riboldi, Giulia Mornati, Massimo Molteni, Chiara Cantiani

**Affiliations:** 1 Child Psychopathology Unit, Scientific Institute IRCCS E. Medea, Bosisio Parini, 23842 Lecco, Italy; elenamaria.riboldi@lanostrafamiglia.it (E.M.R.); g.mornati1@campus.unimib.it (G.M.); massimo.molteni@lanostrafamiglia.it (M.M.); chiara.cantiani@lanostrafamiglia.it (C.C.); 2 Centre for Addiction and Mental Health (CAMH), University of Toronto, Toronto, ON M6J 1H4, Canada; cecilia.marino@utoronto.ca; 3 Bioengineering Lab, Scientific Institute IRCCS E. Medea, Bosisio Parini, 23842 Lecco, Italy; caterina.piazza@lanostrafamiglia.it

**Keywords:** autism spectrum disorder, infants, frontal EEG alpha asymmetry, early detection

## Abstract

Previous research found that the parental autism phenotype is associated with child autism spectrum disorder (ASD), even if the pathway between autistic traits in parents and child ASD is still largely unknown. Several studies investigated frontal asymmetry in alpha oscillation (FAA) as an early marker for ASD. However, no study has examined the mediational effect of FAA between parental autistic traits and child ASD symptoms in the general population. We carried out a prospective study of 103 typically developing infants and measured FAA as a mediator between both maternal and paternal autistic traits and child ASD traits. We recorded infant baseline electroencephalogram (EEG) at 6 months of age. Child ASD symptoms were measured at age 24 months by the Child Behavior Checklist 1½–5 Pervasive Developmental Problems Scale, and parental autistic traits were scored by the Autism spectrum Quotient questionnaire. The mediation model showed that paternal vs. maternal autistic traits are associated with greater left FAA which, in turn, is associated with more child ASD traits with a significant indirect effect only in female infants vs. male infants. Our findings show a potential cascade of effects whereby paternal autistic traits drive EEG markers contributing to ASD risk.

## 1. Introduction

Autism spectrum disorder (ASD) is a complex and heterogeneous condition characterized by social communication deficits and repetitive patterns of behavior [1]. Twin studies show that ASD is a heritable condition, with heritability estimates ranging between 64% and 91% [2]. Genetic susceptibility appears to be expressed in relatives of individuals with ASD through an independent segregation of a broader range of subclinical features (autistic traits) in social communication and atypical patterns that are referred to as representing the broader autism phenotype (BAP). Several studies demonstrated that autistic traits are distributed normally in the general population and are heritable [3]. In particular, parental autistic traits have been found associated with child ASD symptoms in both ASD samples and the general population, suggesting that broader autistic traits are important to identify both clinical and subclinical conditions [3]. However, even if BAP and ASD seem to exist on a continuum, it is still unknown whether and how maternal and paternal autistic traits are differentially associated with child social communication development. Several studies examined sex-specific associations between parental autistic traits and child ASD symptoms [4,5,6]. For example, Schwichtenberg et al. [5] found that paternal autistic traits predicted child ASD severity, while this relationship was not found for mothers. Klusek et al. [7] showed that paternal autistic traits (rigid and untactful traits) were associated with more social deficits in their ASD children. Conversely, a significant association emerged between child performance on a facial identity recognition task and maternal autistic traits, with no relationship between fathers and their children’s scores [6]. Overall, the idea that paternal characteristics are more strongly associated with child ASD phenotype than maternal characteristics is more consistent with the literature, but it has not been well replicated.

Parental autistic traits are also relevant for the evaluation of endophenotypes [8]. A study using an eye-tracker system in 8-month-old infants found that paternal autistic traits were associated with infants’ attentional functioning, suggesting that early impairments in low-level attentional systems may affect high-level social impairment [9].

Although it has been clearly established that child ASD traits can be influenced by parental autistic traits, the underlying mechanisms and the pathway between parental autistic traits and ASD symptoms in children are still largely unknown. Previous electroencephalogram (EEG) studies showed that anomalous oscillatory organization in multiple frequency bands was strongly associated with ASD, with many of these studies emphasizing the crucial role of individual changes in frontal EEG alpha power [10]. Alpha-band oscillations are associated with precise timing of sensory and cognitive inhibition [11]. Interestingly, significant differences in baseline alpha power were identified in infants at high risk for ASD (siblings of children with ASD) compared to typically developing infants, whereby high-risk infants at age 6 months showed lower alpha power as compared to controls [11].

In addition to spectral power differences, changes have been reported in hemispheric asymmetry of the frontal alpha band. Frontal alpha asymmetry (FAA) refers to the difference in EEG power between the frontal right hemisphere and the frontal left hemisphere [12]. Differences in alpha activity between the left and right hemispheres have been used to measure neural activity as a metrics for frontal lobe organization. In particular, because alpha power is inversely related to cortical activation (meaning that decreased alpha power reflects greater brain activity), right FAA indicates higher cortical activation in the right hemisphere and left FAA indicate higher cortical activation in the left hemisphere. In other words, positive values are associated to left FAA (left hemisphere activation) and negative values are associated to right FAA (right hemisphere activation).

FAA has been found associated with ASD [13,14]. Sutton and colleagues [14] examined the relationship between FAA, social impairment, and social anxiety in a sample of high-functioning ASD children compared to controls. The groups significantly differed on FAA, social impairment, and anxiety symptoms. ASD children with right FAA displayed more social deficits and ASD symptoms, whereas greater left FAA was associated with less social deficits and more anxiety symptoms.

Interestingly, research with typically developing infants has provided evidence that FAA changes during early years of life. Typically developing infants gradually shift from right FAA at age 6 months to left FAA at age 18 months [15,16,17,18]. Interestingly, recent evidence indicates that 6-month-old infants at high risk for ASD show an opposite developmental trajectory in FAA, shifting from left FAA at age 6 months to right FAA by age 18 months [17]. Overall, these data demonstrated a different hemispheric organization in infants at high risk for ASD, whereby frontal asymmetry may represent one of the earliest potential endophenotypes for ASD. Despite the fact that FAA has been found associated with ASD [16,17] and parental autistic traits are associated with child ASD [8], no study has explored the role of autistic traits in mothers and fathers on FAA and later child ASD symptoms concurrently.

In addition, recent growing evidence suggests that the pattern of frontal EEG asymmetry might be associated with psychological processes in female infants to a greater extent than male infants. Indeed, there is strong evidence that the link between FAA and psychosocial difficulties may be moderated by sex, with stronger associations in female infants than male infants [19,20]. Even if there are no studies in the ASD field, it may be interesting to explore the relationship between FAA and ASD symptoms in female infants and male infants separately.

Based on this, we conducted a prospective longitudinal study on 103 typically developing infants (general population) and investigated FAA at age six months as a possible mediator of the impact of parental autistic traits on child ASD symptoms. The study aimed to determine 1) whether parental autistic traits are associated with FAA at age six months and child ASD symptoms at age 24 months 2) whether six-month FAA may reflect a potential neural mechanism predicting child ASD symptoms at age 24 months, 3) whether 6-month FAA is a mediator of the contribution of parental autistic traits to ASD-related symptoms, and 4) whether child sex moderates the associations between parental autistic traits, FAA, and ASD symptoms. Our assumption was that FAA significantly predicts child ASD traits and that FAA would serve as a potential neural mediator between parental autistic traits and child ASD-outcome. Based on previous research indicating the influence of sex on the FAA link to developmental psychopathology, we hypothesized that the tested mediation model is moderated by child sex, with a stronger effect in female infants than male infants.

## 2. Materials and Methods

### 2.1. Sample

At 6 months of age, 103 typically developing infants took part in the study (female-to-male ratio = 0.51). The sample was recruited from two hospitals in Northern Italy [21,22]. Inclusion criteria were (a) gestational age ≥36, (b) birth weight ≥2500 g, (c) Bayley Cognitive Score at 6 months ≥7 [23], and (d) no certified intellectual disabilities among first-degree relatives. Families with a diagnosis of ASD among first-degree relatives were also excluded, since we decided to focus on a broad autism phenotype in the general population. Descriptive statistics of demographics and clinical characteristics are shown in Table 1. Parents of all children had given their informed consent for inclusion before participation in the study. The study was conducted in accordance with the Declaration of Helsinki, and the protocol was approved by the Ethics Committee of IRCCS Medea (Ricerca Corrente “2016, 2017, 2018, 2019“, Ricerca Finalizzata “NET-2013-02355263-2” and “5 per mille” funds for biomedical research).

### 2.2. Frontal EEG Alpha Asymmetry

#### 2.2.1. EEG Data Acquisition

Four minutes of baseline EEG at age 6 months (M = 6.46 months; SD = 0.49) were used to compute alpha asymmetry scores. Baseline EEGs were recorded after an experimental session (i.e., a passive oddball paradigm intended to test auditory processing skills; see [27]. During EEG recording, infants were looking at an experimenter blowing soap bubbles.

#### 2.2.2. EEG Data Processing and Analysis

EEGs were recorded from 60 scalp electrodes using HydroCel Geodesic sensor nets (Electrical Geodesics, Inc., Eugene, Oregon, USA). The vertex electrode was the online reference. EEGs were recorded with a sampling rate of 250 Hz and an online band-pass filter (0.1–100 Hz). After recording, EEGs were exported and further processed using lab-internal MATLAB (Mathworks, Natick, MA, USA) routines and the EEGLAB toolbox [28]. Data were band-pass filtered at 1–47 Hz. Bad channels were identified by means of the EEGLAB “TrimOutlier” plugin (identification criteria: 5 < all channels SD < 100) and interpolated with a spherical spline (a maximum of 12 out of 60 channels were interpolated, M = 3.3, SD = 2.7). Data were then re-referenced offline to an average reference and segmented in one-second non-overlapping epochs. Bad EEG epochs were identified and rejected using two EEGLAB functions: (1) “find abnormal values”, marking for rejection epochs in which EEG values exceeded ±150 μV) and (2) “find abnormal trends”, marking for rejection epochs corrupted by linear drift (setting parameters: R = 0.3, max slope = 150 μV). Additional manual artifact inspection was computed. A minimum of 60 artifact-free segments (M = 119.8; SD = 39.9) was used for subsequent power analysis. Power spectral density (PSD) was estimated by Welch’s method [26,29] with non-overlapping 0.5 s windows. PSD values were calculated for each channel in each epoch and then averaged across segments. Following previous literature [17,30], the mean power in the infant alpha frequency band (6–9 Hz) was computed. We selected two clusters of electrodes (based on and adapted from [17], frontal left hemisphere: 9, 11, 12, 13, 14 and frontal right hemisphere: 2, 3, 57, 59, 60 (see Supplementary Materials) and power values were averaged across electrodes within each cluster. In full-spectrum data, we focused on frontal alpha asymmetry (FAA) that has been well characterized in infants. Frontal asymmetry scores were calculated from log-transformed PSD values in selected clusters as follows: (right − left)/(right + left). This formula has been used in most studies to summarize the relative activity at homologous right and left sites [31]. Use of this formula to calculate FAA offers the advantage of minimizing bias due to individual differences in skull thickness that might influence the power spectrum amplitude. In addition, it approaches a normal distribution and shows good stability and reliability [32]. Positive values indicate left FAA and negative values indicate right FAA (M = 0.03; SD = 0.13).

#### 2.2.3. Autistic Traits in Parents: The Autism Spectrum Quotient

The Autism Spectrum Quotient (AQ) is a self-administered questionnaire to quantify autistic traits in the general population [25], with the Italian version by [33]. The AQ questionnaire offers several advantages, including subscales tapping both social and non-social aspects of behavior and cognition and a brief, self-administered, and forced-choice format [34]. Subjects are instructed to respond to each of the 50 items using a 4-point Likert scale as follows: “definitely agree”, “slightly agree”, “slightly disagree”, and “definitely disagree”. The maximum score on the AQ is 50 points, with higher scores meaning higher presence of autistic traits. Two cut-offs were previously described/reported [25]: clinical threshold (raw scores ≥ 32) and screening cut-off (raw scores ≥ 26). Reflecting the non-clinical nature of our sample, only 3 parents (all fathers) reached the clinical threshold and only 13 parents (9 fathers and 4 mothers) reached the screening cut-off. The AQ questionnaire was completed by both parents upon their children’s inclusion in the study. Total AQ scores were transformed into *z*-scores (see Table 1) and were used in further analysis.

#### 2.2.4. Autistic Traits in Infants: The CBCL 1½–5 Pervasive Developmental Problems Scale

The Child Behavior Check List 1½–5 (CBCL 1½–5) consists of 99 items designed to rate emotional and behavioral problems in toddlers. Items are scored by parents on a 3–point Likert scale (0 = not true; 1 = sometimes true; 2 = very true) and they refer to a time span of 6 months before the questionnaire completion. In our sample, 73 questionnaires were filled by mothers (80%), 5 by fathers (5%), and 14 by both parents (15%). This measure with strong psychometric properties across cultures has been translated into, and validated in Italian [35,36]. For the purpose of this study, the Pervasive Developmental Problems scale (PDP) was used as child ASD symptoms and PDP T-scores (mean = 50; SD = 10) were used in the analysis. Reflecting the non-clinical nature of our sample, only 7 children (4 female infants and 3 male infants) reached the clinical threshold (T ≥ 65).

### 2.3. Statistical Analysis

Descriptive statistics and Pearson’s bivariate correlations to examine the associations among study variables were run using SPSS, Version 25.0 (IBM Corp., Armonk, NY, USA). The association between FAA and ASD-related traits was assessed by linear regression analysis: the CBCL 1½–5 PDP score was entered as the dependent variable and FAA was set as the predictor.

To investigate the contribution of paternal and maternal AQ to ASD-related traits in children and the potential role of FAA as a mediator, we used Structural Equation Modeling (SEM) [36], as implemented in the MPLUS software (Los Angeles, CA) [37]. SEM simultaneously models all paths, providing a more accurate estimation of mediation effects [38,39] than more traditional tests based on sequential regressions.

The mediation model tested the hypothesis that ASD-related traits in children would be explained by a sequence of potentially associated effects involving parental autistic traits and FAA. Specifically, the following model was proposed: maternal and paternal AQ → FAA → child ASD traits. We then assessed the mediation model which best described the associations between the measured variables [39]. Finally, moderation by child sex was examined to assess whether relations between study variables differed by sex (male vs. female).

The bias-corrected 5000 bootstrap technique was used to test mediation effects [36]. Confidence intervals (95% CI) that do not contain zero indicate significant indirect effects [40,41,42,43]. Several fit indices are used to assess the best fitting model: a) Chi-Square assessing the difference in magnitude between the model estimated variance/covariance matrices, b) the RMSEA (root mean square error of approximation) considering the complexity of the model [42], c) the SRMR (standardized root mean square residual) indicating the average residual value from the model fit covariance matrix to the sample covariance matrix, and d) the CFI (comparative fit index) indicating the improvement in overall model fit by comparing the hypothesized model with a more restricted one, which specifies no relations among variables. RMSEA ≤ 0.05, SRMR ≤ 0.08, and CFI ≥ 0.95 indicate a good model fit [42,43,44,45]. To allow for the use of all available data with inclusion of subjects with missing data, we considered the full information maximum likelihood estimation. Significant effects were set to *p*-values ≤ 0.05.

## 3. Results

We first examined the correlations between maternal and paternal AQ (*z*-scores), CBCL 1½–5 Pervasive Developmental Problems (T-scores), and FAA. We found a significant correlation between FAA and CBCL 1½–5 PDP scores (*r* = 0.42, *p* < 0.001), with greater left FAA being associated with more child ASD-related symptoms. Correlations between paternal AQ scores and FAA (*r* = −0.29, *p* = 0.003) and paternal AQ scores and CBCL 1½–5 PDP scores were low-to-moderate (*r* = −0.39, *p* < 0.001). Higher paternal autistic traits (negative z-scores for AQ values) were associated with greater left FAA at age 6 months and more child ASD-related symptoms at age 24 months. No significant correlation emerged between maternal AQ scores and FAA (*r* = −0.11, *p* = 0.244) and a low—although not significant—correlation was found between maternal and paternal AQ scores (*r* = 0.14, *p* = 0.157). Finally, no significant correlation was found between maternal AQ scores and CBCL 1½–5 PDP scores (*r* = −0.09, *p* = 0.394).

### 3.1. Testing a Mediation Model: FAA as a Mediator between Parental AQ Scores and Child ASD-Related Traits

**Figure 1:**
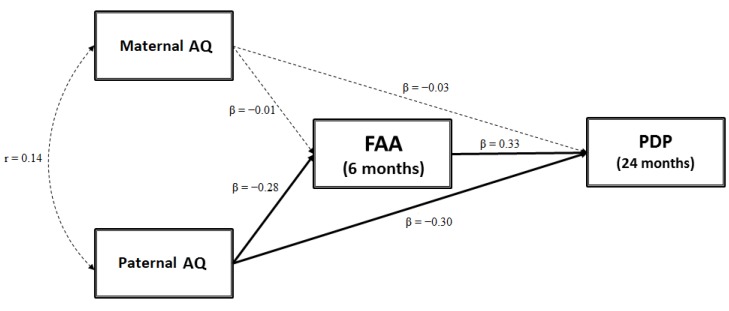
Frontal asymmetry in alpha oscillation (FAA) as a mediator between maternal and paternal autism spectrum quotient (AQ) scores and child autism spectrum disorder (ASD)-related traits. PDP—Pervasive Developmental Problems.

After carrying out descriptive and correlational statistics, we used SEM to test the mediation model shown in Figure 1, which assumes that maternal and paternal AQ scores contribute to FAA. FAA, in turn, affects child ASD-related traits. The model provided a good fit to the data (χ^2^ (5) = 37.31, *p* < 0.001; RMSEA = 0.000, CI (90%) = 0.000–0.000; CFI = 1.00; SRMR = 0.000) and accounted for 26.3% of the variance in CBCL 1½-5 PDP scores. Figure 1 shows standardized coefficient estimates. The mediation model yielded several significant direct effects. There was a significant path coefficient from paternal AQ to CBCL 1½–5 PDP scores (β = −0.30, *p* = 0.004). Children with higher paternal autistic traits showed higher PDP scores at age 24 months. No significant association from maternal AQ to CBCL 1½–5 PDP scores emerged (β = −0.03, *p* = 0.719). Significant effects were found from paternal AQ scores to FAA (β = −0.28, *p* = 0.014), and from FAA to CBCL 1½–5 PDP scores (β = 0.33, *p* = 0.028): Higher paternal autistic traits predict greater left FAA and greater left FAA predicts higher infant ASD-related traits. However, 5000 bootstrap estimates (95% CI) showed that the indirect effect from paternal AQ to CBCL 1½–5 PDP scores via FAA was not significant (β = −0.092; SE = 0.256; 95% CI (−0.193; 0.010), *p* = 0.112).

### 3.2. Testing a Moderated Mediation Model: FAA as a Mediator between Parental AQ and Child ASD-Related Traits Moderated by Sex

**Figure 2:**
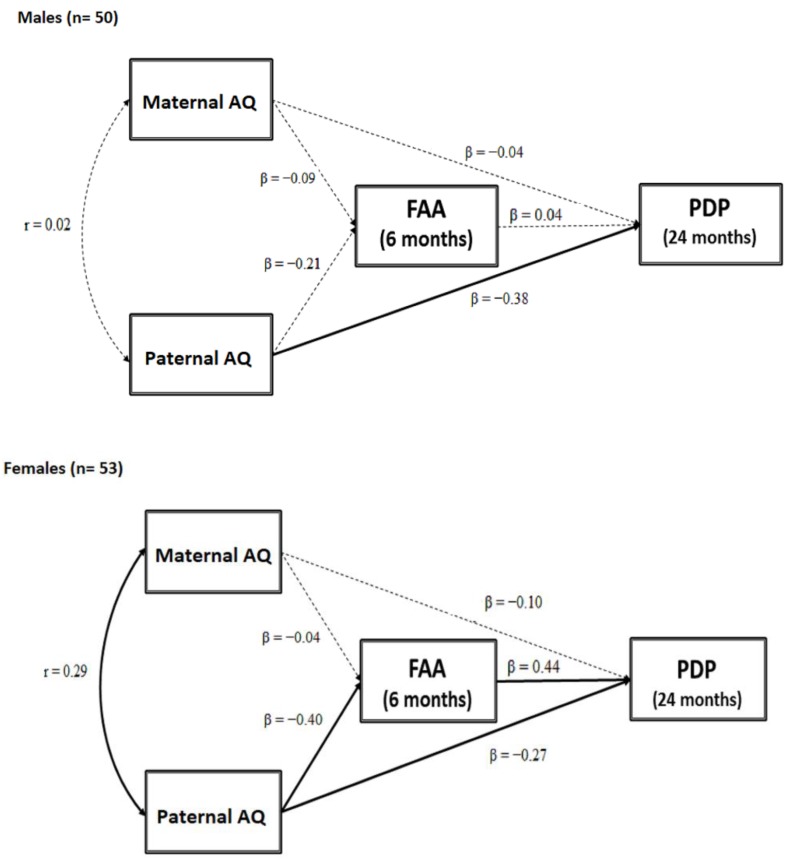
FAA as a mediator between maternal and paternal AQ and child ASD-related traits moderated by sex.

Moderated mediation was also applied to examine whether child sex moderated the associations between maternal and parental AQ scores, FAA, and child ASD-related outcome via multigroup analyses in the SEM framework (see Figure 2 for group-specific parameter estimates).The model provided a good fit to the data (χ^2^ (10) = 44.74, *p* < 0.001; RMSEA = 0.000, CI (90%) = 0.000–0.000; CFI = 1.00; SRMR = 0.000) and accounted for 15.5% of the variance in the CBCL 1½–5 PDP scores in male infants and 40.5% of the variance in the CBCL 1½–5 PDP scores in female infants.

Standardized estimates of path coefficients in each group are depicted in Figure 2. In male infants, there was only a direct effect of paternal AQ to CBCL 1½–5 PDP scores (β = -0.38, *p* = 0.027), with higher paternal autistic traits predicting higher CBCL 1½–5 PDP scores. No other direct or indirect effect was found.

In female infants, significant associations were found from paternal AQ scores and FAA (β = −0.40, *p* = 0.010), from paternal AQ scores and CBCL 1½–5 PDP (β = −0.27, *p* = 0.038) and from FAA to CBCL 1½–5 PDP scores (β = 0.44, *p* = 0.014): Higher paternal autistic traits predicted greater left FAA which, in turn, predicted higher CBCL 1½–5 PDP scores. A significant correlation between maternal and paternal AQ scores (*r* = 0.29; *p* = 0.041) was also found.

**Figure 3:**
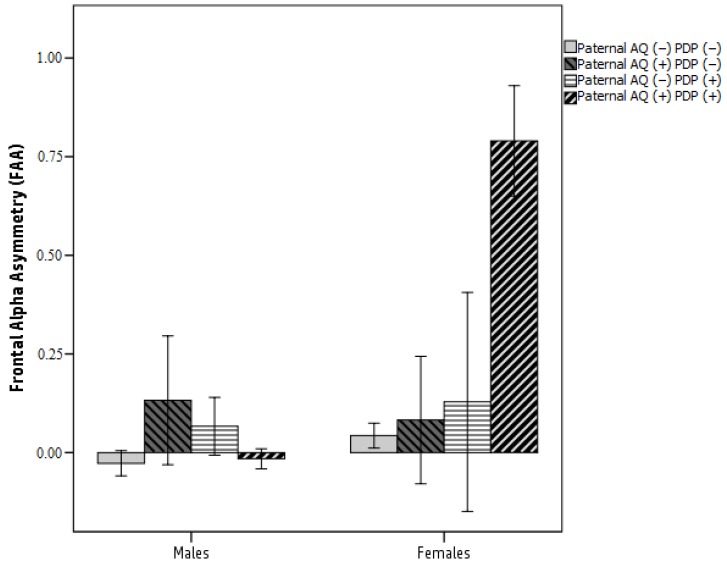
FAA differences between paternal AQ scores, CBCL 1½−5 PDP scores and child sex. Legend: Paternal AQ scores (−): low paternal autistic traits; Paternal AQ scores (+): high paternal autistic traits; PDP scores (−): low CBCL 1½–5 PDP scores; PDP scores (+): high CBCL 1½−5 PDP scores.

Interestingly, 5000 bootstrap estimates (CI 95%) showed that the indirect effect from paternal AQ to CBCL 1½–5 PDP scores via FAA was significant for female infants (β = −0.176; SE = 0.579; 95% CI (−0.334; −0.016), *p* = 0.041) but not for male infants (β = −0.008; SE = 0.117; 95% CI (−0.251; 0.246), *p* = 0.815). In female infants, paternal AQ scores were associated with greater left FAA at age 6 months, which affected child ASD-related traits at age 24 months. In other words, FAA mediates the association between paternal AQ scores and child ASD-related symptoms, and this link was moderated by child sex in female infants but not in male infants (for graphical purposes only, see Figure 3).

## 4. Discussion

This is the first general population study looking at frontal asymmetry in EEG alpha oscillation at age 6 months as a potential mediator in the developmental pathway from maternal and paternal autistic traits to child ASD-related traits at age 24 months.

### 4.1. Parental Autistic Traits and Child ASD Symptoms

Not surprisingly, paternal autistic traits were associated with more child ASD-related symptoms, supporting the assumption that broader autistic traits in parents may be useful in identifying both clinical and subclinical conditions [3]. This finding is well replicated in previous studies. The association between higher autistic traits in parents and their children strongly supports an underlying genetic mechanism [46]: Shared genetic variability may be a plausible pathway for familial transmission of common factors between parental autistic traits and their child ASD-related traits. Consistent with the literature, we found that paternal characteristics are associated with child ASD phenotype rather than maternal characteristics [4,5,6], supporting greater patrilineal effects within families [7].

### 4.2. Parental Autistic Traits and Frontal Asymmetry in Alpha Oscillation

Our findings also showed that higher paternal autistic traits were associated with greater left FAA at age 6 months. Typically developing infants shifted from greater right FAA (right frontal activation) at age 6 months to relative greater left FAA (left frontal activation) at age 18 months [15,16]. Interestingly, an opposite pattern was found in infants at high risk for ASD (siblings of children with ASD) from greater left FAA at age 6 months to greater right FAA at age 18 months [17,18,47]. In line with this piece of evidence, we found that at-risk infants (having a parent with higher autistic traits) aged 6 months showed greater left FAA, suggesting an atypical organization and lateralization of oscillatory processes. We might speculate that hemispheric organization follows a different developmental shift in infants with higher paternal autistic traits. However, since EEG measures at age 18 months were not obtained, further studies are needed to confirm this hypothesis.

### 4.3. Frontal Asymmetry in Alpha Oscillation and Child ASD Symptoms

Greater left FAA at age 6 months was associated with increased child ASD-related traits at age 24 months. Past research has linked FAA to different cognitive and behavioral processes, supporting the role of FAA as a biomarker for psychopathology [48]. FAA is a measure of the propensity to adopt an “approach or withdrawal” behavior [48], with greater left frontal activity associated with an increased tendency to approach, and greater right frontal activity associated with an increased tendency to withdraw. Taken together, frontal alpha asymmetry can be interpreted with respect to the amount of motivation towards (approach) or away from (withdraw) something or someone. Relating to ASD, greater right frontal asymmetry is associated with social impairment and earlier onset of ASD symptoms [13,14], whereas less social impairment has been observed in children with greater left frontal asymmetry.

However, the direction of the effect that we found in the present study (i.e., greater left FAA at age 6 months associated with earlier ASD-related symptoms) was different from what was expected based on previous literature. This discrepancy could be due to different population characteristics (infants vs. children/adolescents; typically developing infants vs. high-functioning ASD children). Since frontal asymmetry tends to change over the first two years of life, with a shift in lateralization from right to left FAA in typically developing infants and from left to right FAA in infants at risk for ASD [15], the different direction of the reported effects between the present and previous studies might be due to maturation effects. This needs to be confirmed in more overlapping study populations.

### 4.4. Frontal Asymmetry in Alpha Oscillation as a Mediator between Paternal Autistic Traits and Child ASD Symptoms

Perhaps more importantly, we found that paternal, but not maternal, autistic traits are directly associated with FAA and FAA directly affects child ASD-related traits. This different association may reflect different biological mechanisms based on parent-of-origin effects, namely the genetic effects on the (endo)phenotype of an offspring that are dependent on the parental origin of the associated genetic variants. Several studies [49,50] found that parents may transmit genes or epigenetic dysregulation affecting ASD through sex-specific pathways and, in line with our own results, parent-of-origin effects were found in ASD, with a paternal over-transmission of risk alleles for ASD [49]. Even if further research is needed, our results may support the importance to explore the role of epigenetic modulators in the etiology of ASD. If parent-of-origin effects are proven, more homogeneous ASD-related phenotypes could be identified by grouping according to parental ASD traits.

Although in an exploratory manner, we found a significant indirect effect and provided the first evidence that FAA at age 6 months significantly mediates the contribution of paternal autistic traits to ASD-related traits in their children, while this is seen at age 24 months in female infants but not in male infants. It is well replicated that male infants are more frequently diagnosed with ASD than female infants, with a reported sex ratio of 4:1 [51]. Sex differences may reflect the distinctive sexual dimorphism of the brain, including hormonal and structural factors as well as genetic and epigenetic influences, which emerge during development. For example, effects of serotonin genotypes on EEG activity were found to vary as a function of sex. The 5-HTTLPR polymorphism was associated with modulation of the EEG activity at different EEG frequencies only in female infants and not in male infants [52], suggesting that baseline EEG frontal activity marks different neurobiological processes in female infants and male infants. Understanding the mechanisms underlying the sex difference in ASD is not only fundamental per se, but it might crucially contribute to unravelling the well-known sex differences in prevalence, age of onset, and severity that we observe in many psychiatric diseases, including depression and anxiety disorder and ADHD, in which a role of FAA has been reported [20,53]. Even if replication studies are necessary, it is conceivable that FAA involved in cortical development—if combined with higher parental autistic traits—could potentiate different genetic vulnerabilities in male infants and female infants, specifically ASD-related problems.

This study presents some limitations. First, ASD traits were assessed solely by parental report. Although no evidence for report bias regarding parent–offspring autistic traits emerged in previous studies [54], in our study we cannot exclude that parental ASD traits might have an effect on parental perception of their child’s behavior. Therefore, we suggest that future studies should focus on direct assessment of autism-related symptoms. Second, we measured EEG frontal alpha asymmetry only at 6 months of age. As reported by previous studies [15,16,17], FAA seems to be developmentally sensitive from age 6 to 24 months. Future longitudinal studies with larger samples of typically developing and at-risk infants are important to increase our confidence on (a) typical EEG asymmetry trajectories and how such EEG trajectories relate to different broader autism phenotype domains. A further point to be highlighted concerns the specificity of the relationship between FAA and ASD traits. Previous studies reported that oscillations in different frequency bands are related to the development of other cognitive skills (i.e., oscillations in the gamma frequency bands have been reported to be predictive of language skills) [55]. In our study, we tried to disentangle a possible connection between frontal alpha asymmetry and language by means of exploratory correlations with language measures at 24 months and found—as expected—no significant correlations, thus supporting the assumption of a specific pathway between FAA and ASD traits. Further studies are needed in this direction.

## 5. Conclusions

These findings support the use of objective measurements of EEG frontal alpha asymmetry to delineate specific pathophysiological mechanisms in ASD. Notably, this study reports a prediction of ASD symptoms at age 24 months. However, our longitudinal data collection is ongoing, and we are prospectively following our current cohort to identify children who will or will not receive a diagnosis of ASD. Characterization of reliable biomarkers will guide the detection of the most vulnerable infants that will benefit from early intervention and rehabilitation, with the long-term aim of substantially reducing the heavy impact of ASD on the National Health System.

## Figures and Tables

**Table 1:** Descriptive statistics of demographics and clinical characteristics.

	Total Sample (*n* = 103)	Males (*n* = 50)	Females (*n* = 53)
	Mean (±SD)	Mean (±SD)	Mean (±SD)
Birthweight (grams)	3252.20 (±468.08)	3325.33 (±481.35)	3179.07 (±453.03)
Gestational age (weeks)	39.08 (±1.47)	39.67 (±1.27)	38.49 (±1.66)
Maternal educational level ^a^	58.04 (±17.05)	56.50 (±19.39)	59.52 (±14.49)
Paternal educational level ^a^	49.31 (±17.68)	50.41 (±17.67)	48.27 (±17.79)
Socioeconomic status ^b^	61.47 (±15.51)	61.10 (±15.63)	61.83 (±15.53)
Bayley cognitive subscale at 6 months ^c^	12.07 (±1.81)	11.82 (±1.96)	12.30 (±1.65)
Paternal AQ (raw scores)	17.81 (±6.22)	19.20 (±6.49)	16.44 (±5.68)
Maternal AQ (raw scores)	14.49 (±5.77)	15.28 (±5.95)	13.73 (±5.53)
Paternal AQ ^d^	−0.26 (±1.37)	−0.53 (±1.40)	0.003 (±1.22)
Maternal AQ ^d^	0.03 (±1.09)	−0.03 (±1.16)	0.10 (±1.02)
CBCL 1½-5 Pervasive Developmental Problems ^e^	53.42 (±5.94)	52.84 (±4.88)	53.98 (±6.81)

^a^ The educational level of mothers and fathers was scored on a 9-point ordinal scale created ad-hoc and based on the Italian school system. ^b^ Socioeconomic status was scored according to Hollingshead 9-point scale, whereby a score ranging from 10 to 90 was assigned to each parental job and the higher of two scores was considered when both parents were employed [24]. ^c^ Age-standardized (mean = 10; SD = 3) score on the Bayley cognitive subscale [23]. ^d^ Age-standardized *z*-scores (mean = 0; SD = 1) for the total Autism Spectrum Quotient (AQ) score [25]. ^e^ Age-standardized T-scores (mean = 50; SD = 10) for the Child Behavior Check List 1½–5 (CBCL 1½–5) [26].

## Supplementary Materials

The following are available online at https://www.mdpi.com/2076-3425/9/12/342/s1, Figure S1: Sensor layout of the 60-channel Hydro-Cel Geodesic Sensor Net used in the study. Red and blue squares represent the electrodes included, respectively, in the Left and Right frontal clusters and entered into statistical analyses (see Gabard-Durnam et al., 2015).
