# Supplementary material for: Paternal—but Not Maternal—Autistic Traits Predict Frontal EEG Alpha Asymmetry in Infants with Later Symptoms of Autism
Source: Brain Sci. 2019 Nov 26;9(12):342. doi: 10.3390/brainsci9120342 (PMC6956226; doi:10.3390/brainsci9120342)
Supplement: Supplementary file 1 [file brainsci-09-00342-s001.pdf]

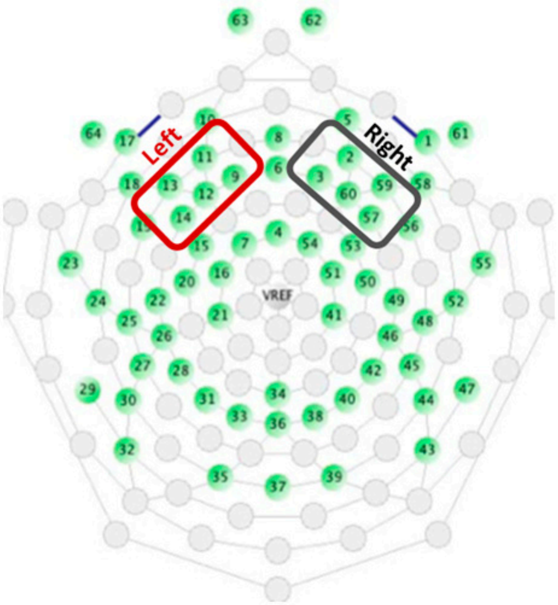

Supplementary Figure S1 Sensor layout of the 60-channel Hydro-Cel Geodesic Sensor Net used in the study. Red and blue squares represent the electrodes included, respectively, in the Left and Right frontal clusters and submitted to statistical analyses (see Gabard-Durnam et al., 2015)
